# Clinical utility of chromatin analysis

**DOI:** 10.18632/oncotarget.25991

**Published:** 2018-08-21

**Authors:** Andreas Kleppe, Håvard E. Danielsen

**Affiliations:** Håvard E. Danielsen: Institute for Cancer Genetics and Informatics, Oslo University Hospital, Oslo, Norway; Department of Informatics, University of Oslo, Oslo, Norway; Nuffield Division of Clinical Laboratory Sciences, University of Oxford, Oxford, United Kingdom

**Keywords:** digital pathology, clinical utility, chromatin analysis, nucleotyping, pan-cancer

Epigenetics, the heritable traits not coded in the DNA sequence, determines gene expression through mechanisms such as DNA methylation and histone modifications [1]. Higher-order chromatin organisation has been shown to alter during cell differentiation [2] and explains much of the variation in regional mutation rates in cancer cells [3].

Different attributes of higher-order chromatin organisation can be computed from bright-field microscopy images of tumour cell nuclei stained with a DNA-specific stain, and the relation between such characteristics and cancer prognosis has been studied for a few decades. Our own analyses suggest that the entropy of the chromatin structure in thousands of tumour cell nuclei indicates the clinical outcome of the patient. Despite much research and many promising preliminary findings, none had independently validated the methodology in external cohorts, which is necessary to obtain realistic estimates of the prognostic capabilities. We therefore developed an objective, dichotomous marker based on automatic estimation of chromatin entropy, termed Nucleotyping and marker of chromatin heterogeneity, and designed it to be particularly resilient to vagaries of the measurement process [4]. A cohort of 390 patients treated for stage I or II colorectal cancer (CRC) at the Aker University Hospital in Oslo, Norway, was utilised in the discovery phase, and six external cohorts comprising several cancer types were used for independent validations, both to reliably assess the ability of Nucleotyping to predict cancer-specific survival (CSS) and to investigate whether chromatin heterogeneity is a tumour characteristic common for multiple tumour entities [4].

Analysis of 442 stage I or II CRC patients from the Gloucester Colorectal Cancer Study, UK, replicated the findings from the discovery cohort both in terms of Nucleotyping’s hazard ratio (HR) in univariable and multivariable analyses (about 1.8) and 5-year CSS of stage II patients (83-85% if chromatin homogeneous [CHO] and 72% if chromatin heterogeneous [CHE]). Only 3% of these patients received adjuvant treatment and excluding these from the analyses did not substantially change the results. Slightly larger HRs (about 2.4) were observed in analysis of 441 patients with stage II CRC from the QUASAR 2 trial, all of which after surgery received capecitabine with or without bevacizumab. Adjuvant chemotherapy has been shown to increase survival in stage II CRC, but the absolute improvement is small, indicating the need for accurate identification of high-risk patients which benefit most from the treatment, at least in absolute terms [5, 6]. Nucleotyping risk stratified stage II CRC patients more precisely than microsatellite instability status and correlated weakly or not at all with established clinical and pathological markers which are often used to identify high-risk stage II CRC patients, suggesting that chromatin heterogeneity could enhance the identification of high-risk patients and thereby possibly the selection of patients for adjuvant chemotherapy.

The European Society for Medical Oncology (ESMO) recommends adjuvant chemotherapy for early-stage ovarian carcinoma patients who were suboptimally staged or at higher risk of recurrence [7], and exploratory analyses in stage I suggest most benefit for clinically high-risk patients, defined as clear cell, grade 3 or stage IB/IC grade 2 [8]. Nucleotyping predicted CSS in univariable and multivariable analyses of 246 patients with stage I ovarian carcinoma, and in the subgroup of clinically high-risk patients (HR 1.9, 95% confidence interval [CI] 1.1-3.2; *p* = 0.015). Integrating Nucleotyping with pathological evaluations may improve risk stratification in early-stage ovarian carcinoma, and randomised trials are warranted to assess the survival benefit of adjuvant single-agent or combination chemotherapy in different risk groups.

Uterine sarcoma is a rare and generally aggressive disease without consensus on adjuvant treatment [9]. Nucleotyping depicted 5-year CSS in univariable and multivariable analyses of all assessable cases of uterine sarcoma in Norway between 1970 and 2000, in total 354 patients. In both major histological subtypes and other subtypes combined, the 5-year CSS of patients with CHE tumours were similar (36% for leiomyosarcoma, 33% for endometrial stromal sarcoma, and 32% for others) and significantly shorter than for CHO patients (59%, 80%, and 65%, respectively). It may be reasonable to offer all CHE patients adjuvant chemotherapy, but clinical trials are needed to determine appropriate treatment plans and their impact on survival.

Nucleotyping predicted CSS in univariable and multivariable analyses of curettage specimens from 791 endometrial carcinoma patients in the Molecular Markers in Treatment of Endometrial Cancer (MoMaTEC) trial. Adjuvant treatment is generally recommended, but not always necessary for stage I clinically high-risk patients, defined as stage IB grade 3 endometrioid or stage I non-endometrioid [10]. Of the 98 patients analysed in this subgroup, the 62 (63%) with CHO tumours had a 5-year CSS of 97%, while the 36 (37%) with CHE tumours had a 5-year CSS of 59%. This indicates that Nucleotyping may be clinically useful in selecting patients for adjuvant treatment; in particular, it seems that a major part of stage I clinically high-risk patients can be spared adjuvant treatment and the long-term morbidities following such treatment.

**Figure 1:**
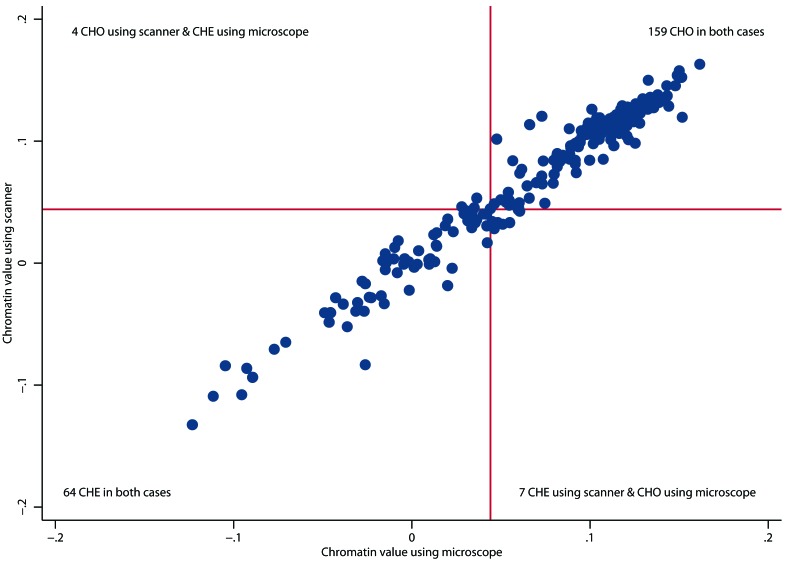
Scatter plot of chromatin value measured using a bright-field microscope and a whole-slide digital scanner The red lines depict the threshold for dichotomising chromatin values; the classification is chromatin heterogeneous (CHE) if the chromatin value is smaller than the threshold and otherwise chromatin homogeneous (CHO). Pearson correlation coefficient was 0.98 (95% confidence interval [CI] 0.97-0.98; *p* < 0.0001) between the chromatin values and 0.89 (95% CI 0.86-0.91; *p* < 0.0001) between the chromatin classifications. Since the microscope was equipped with a 546 nm green filter and a monochrome digital camera while the scanner acquired colour images which were converted to grey scale by averaging, the integrated optical density (IOD) was typically far less in the scanner images and therefore the element width was reduced from 25 to 7.5 in the DNA ploidy histogram computed as a part of the image normalisation method, although the correlation was nearly as good without this adjustment (0.95 between chromatin values and 0.84 between chromatin classifications).

The future of pathology is digital, where microscopes are being replaced by scanners in order to capture whole-slide digital images. To evaluate whether scanner images can be used to reliably assess chromatin heterogeneity, 234 samples of stage II colon tumours were prepared as in our original study [4] and imaged by one of the originally applied microscopes and by a Aperio AT2 scanner (Leica Biosystems, Germany). The nearly perfect correlation between the two measurements (Figure 1) indicates that easier and cheaper clinical implementation of Nucleotyping is possible using scanners.

Chromatin heterogeneity is a tumour characteristic found in many cancer types and indicates shorter CSS independently of most established prognostic markers. There is evidence to suggest that Nucleotyping may be used to guide selection of treatment in several large patient groups. Clinical trials randomised on standard versus indicated treatment are warranted to fully answer how the patients are affected by implementing Nucleotyping in the clinic, in particular with respect to absolute survival benefit and improved quality of life, and full cost-benefit analyses should be performed to estimate the financial implications of utilising this marker to select treatment in the specific patient groups.
